# International Survey on Evaluation and Management of Eosinophilic Esophagitis

**DOI:** 10.1097/WOX.0b013e3182690759

**Published:** 2012-09-15

**Authors:** Christopher C Shaffer, Gisoo Ghaffari

**Affiliations:** 1 Department of Medicine, Pennsylvania State University College of Medicine/Penn State Hershey Medical Center, Hershey, PA; 2 Division of Pulmonary, Allergy, and Critical Care Medicine, Pennsylvania State University College of Medicine/Penn State Hershey Medical Center, Hershey, PA

**Keywords:** eosinophilic esophagitis, evaluation, treatment, educational activities, international, survey

## Abstract

**Background:**

Recommendations regarding evaluation and management of eosinophilic esophagitis (EoE) remain incompletely defined. This survey assesses: how providers across the world diagnose, evaluate, and treat EoE and how educational activities affect management.

**Methods:**

A web-based survey was sent to the members of World Allergy Organization, American College of Allergy, Asthma, and Immunology, and American Academy of Allergy, Asthma, and Immunology. A *χ*^2 ^analysis compared responses based on personal and practice demographics and participation in educational activities.

**Results:**

Of the 200 respondents, 68.5% were from the United States. The majority were allergists, who require biopsy to diagnose EoE, perform allergy testing, and obtain follow-up biopsy after treatment. The following variables had significant differences: (1) US practitioners were more likely to test for immediate-type hypersensitivity to foods and obtain follow-up endoscopic biopsies after the initial treatment; (2) Practitioners encountering patients with EoE more frequently were more likely to ask about personal and family history of atopy, test for immediate-type hypersensitivity to aeroallergens and foods, and recommend follow-up biopsy after treatment; and (3) Practitioners who participate more often in EoE workshops were more likely to perform patch testing for foods, while attendance at EoE lectures increased EoE management confidence.

**Conclusions:**

Diagnostic and management strategies differ based on practice location, EoE patient load, and participation in educational activities. Practitioners who attend more EoE lectures are more confident managing EoE.

## 

Eosinophilic esophagitis (EoE) is a chronic, clinicopathologic inflammatory disease process with an increasing prevalence [1,2]. The prevalence of EoE has been estimated to be 52 per 100,000 patients in the general population and 2.8% of symptomatic patients with dysphagia [3,4]. EoE has been shown to be associated with an impaired quality of life and increased risk of food impaction [5]. Criteria for diagnosis established in the 2007 consensus recommendations were further refined in the 2011 update to the consensus recommendations. The new recommendations establish the nomenclature of EoE and the concept of chronic eosinophilic inflammation. Although the findings of ≥ 15 eosinophils per high-power field on esophageal biopsy as a diagnostic criterion remain unchanged, the importance of considering the clinical symptoms and other pathologic findings are emphasized [2].

The recommendations regarding allergic work-up and management are still incompletely defined. Testing for immediate-type hypersensitivity reactions to aeroallergens has been recommended since 2007 based on studies demonstrating that pulmonary exposure to aeroallergens induces EoE and epithelial hyperplasia [6-15]. Skin prick testing (SPT) for foods to identify potential allergens has been recommended since the initial publication as well [6,16]. Combination of SPT and atopy patch testing (APT) for foods to increase the identification of food allergies has been suggested by authorities in the field [17,18]. This combination has been shown to have a high negative predictive value (except to milk) and a good positive predictive value to identify the potential foods responsible for symptoms of EoE [19]. The consensus recommendations support dietary therapy by means of elimination diet (antigen removal) or elemental diet (amino acid-based formula) for children but do not provide specific recommendations for adults [2,6]. There has been no clear guidance on whether the elimination diet should be empiric (based on the most common food allergens) or based on SPT and APT results [2,6].

Elimination diets, based on combined results of SPT and APT, or empiric removal of the 6 most common food allergens from the diet, both resulted in a resolution of symptoms in roughly 75% of subjects [20-22]. Both strategies also significantly improved esophageal histology, including inflammation and eosinophil levels [21-23]. An elemental diet similarly resulted in normalization of esophageal eosinophil levels but was more efficacious in alleviating esophageal symptoms than elimination diets [24-26]. In fact, elemental diets resulted in cessation of symptoms in more than 95% of patients, suggesting food allergy as a causative agent for EoE [24]. Elemental diets are not preferred, however, because of their adverse effect on quality of life and increased cost. There are no recommendations on whether dietary therapy should be attempted before medical therapy.

Regarding medical treatment, evidence exists for several therapeutic interventions. Proton pump inhibitors (PPIs) are commonly used early in the disease course, often before EoE diagnosis, to treat symptoms suggestive of gastroesophageal reflux disease (GERD). They may also be used to treat concomitant GERD, to fulfill the diagnostic criteria by ruling out GERD as the cause of symptoms, [6] or to assess for and treat PPI-responsive esophageal eosinophilia [2]. A growing body of literature supports the use of topical swallowed steroids not only to control symptoms but also to improve histologic findings [27-38]. However, guidelines on treatment end points and maintenance medical therapy are lacking. Therapeutic use of cromolyn, leukotriene antagonists, [39] and systemic corticosteroids (except during severe, acute events) are not recommended because of lack of efficacy or side effects [2,6].

There is no clear professional guideline on the frequency of monitoring esophageal histopathology in EoE patients. After diagnosis, repeating esophagogastroduodenoscopy with changes in symptoms, at 4 or more weeks after a change in treatment or after a period of treatment noncompliance, have been proposed [2]. However, without a clear knowledge of the natural course or possible complications of long-term untreated or undertreated EoE, clinical practice likely remains highly individualized.

Given the recent discovery of this disease entity, the variety of approaches to management, the amount of emerging evidence, and the number of unanswered questions, the management of EoE assumedly varies among practitioners. This assertion is supported by surveys of allergists and gastroenterologists in the United States that revealed variability in diagnostic criteria and treatment approach [3,40]. The purpose of our study was to assess how providers across the world diagnose, evaluate, and treat patients with EoE and how education affects their approach to patients with EoE.

## Methods

This cross-sectional study of evaluation and management practices of physicians caring for patients with EoE was performed in survey format. The study was approved by the Institutional Review Board of the Pennsylvania State University College of Medicine/Penn State Hershey Medical Center. A link to a web-based survey was distributed to members of the World Allergy Organization, the American College of Allergy, Asthma, and Immunology, and the American Academy of Allergy, Asthma, and Immunology. Participation was by invitation only, restricted to members of the aforementioned organizations. It was not otherwise limited geographically. The survey remained open from October 2010 to January 2011 to allow sufficient time for an adequate number of responses.

The survey consisted of 24 questions. Basic demographic data included the country of medical practice, gender, primary specialty, subspecialty (if any), number of years in practice, setting of practice (academic or private), practice location (urban/suburban or rural), and number of patients seen per week. Practice-related demographic data specific to EoE included frequency of encounters (daily, weekly, or monthly) with EoE patients, average number of encounters with EoE patients per week, and the most common age-group encountered in patients with EoE.

Other questions focused on the diagnostic evaluation of patients with EoE. Respondents were asked if they think esophageal biopsy is required for diagnosis, how often they ask about personal and/or family history of atopy, how frequently they perform allergy testing in EoE patients, and which types of hypersensitivity testing are offered.

There were several questions focused on therapeutic management of EoE. Respondents were asked how often EoE patients are on PPIs at their initial visit, their choice of initial therapy, and second-line therapy. They were also asked whether they recommend a follow-up biopsy after initiation of treatment.

Questions related to education about EoE included the number of lectures and workshops on EoE attended by the physician in the previous 3 years. Moreover, questions about the type of the workshops attended (including, patch testing for foods, elemental diets, food elimination diets, and food allergy), the physician's degree of confidence in management of EoE, and whether there is a perceived need for further education about EoE were included. Should there be a perceived need for further education, there was a question about the most beneficial education format.

Chi-square analysis was used to compare responses with the questions according to the following independent variables: country of practice, gender, specialty, years in practice, type of practice, location of practice, number of patients seen with EoE, frequency of EoE patients seen, number of lectures, and number of workshops on EoE attended.

## Results

The survey was sent to 2000 individuals. There were 200 respondents to the survey, 137 (68.5%) of whom were from the United States (Table 1). All 6 inhabited continents and 33 countries were represented. After the United States, practitioners from Europe participated the most in the survey, including representatives from Spain (5); Italy (4); the United Kingdom (3); Iceland and Greece (2 each); Austria, Belgium, the Netherlands, Portugal, Slovenia, Sweden, and Switzerland (1 each). Australia was represented twice. The characteristics of respondents are shown in Table 1.

**Table 1:** Background Characteristics of Survey Respondents

Characteristics	Number	Percent
Country (n = 200)		
USA	137	68.5
Non-USA	63	31.5
Gender (n = 195)		
Male	105	54
Female	90	46
Primary specialty (n = 200)		
Pediatrics	96	48
Medicine	60	30
Medicine/pediatrics	22	11
Others	22	11
Subspecialty (n = 194)		
Allergy-immunology	166	86
Allergy only	22	11
Other	6	3
Years in practice (n = 200)		
> 10	105	52.5
≤ 10	95	47.5
Type of practice (n = 185)		
Academic	96	52
Private	89	48
Location of practice (n = 194)		
Urban/suburban	183	94
Rural	11	6
Patients seen per week (n = 200)		
> 30	135	67.5
≤ 30	65	32.5
EoE patient encounter frequency (n = 197)		
Monthly	144	73
Weekly	53	27
EoE patients seen per week (n = 195)		
1	163	83
2-3	21	11
> 3	11	6
Average age of EoE patients (n = 194)		
0-10	74	38
11-30	78	40
> 30	42	22

Nearly half of participants responded that they are practicing in an academic setting and have been in practice for more than 10 years. As for medical specialties, 48% of respondents were trained in pediatrics, 30% in medicine, 11% in combined medicine/pediatrics programs, and 11% listed other specialties, many of which were described as allergy, allergy/immunology, or allergology. This reflects the fact that in many countries other than the United States, allergy/allergology is a primary specialty. When respondents identified a subspecialty, they included allergy and immunology (86%), allergy only (11%), gastroenterology, pulmonology, and allergology.

Regarding patient volume, more than two thirds of respondents (67.5%) see more than 30 patients per week. Frequency of encountering patients with EoE was variable. While 27% of respondents encounter EoE patients on a daily to weekly basis, 73% only encounter them monthly or every few months. Seventeen percent of respondents see more than 1 EoE patient per week, while 6% see more than 3. The majority of respondents report that the most common age group of EoE patients they encounter is younger than 30 years, with 38% being in the first decade of life. Only 22% report that the most common age of EoE patients they see is older than 30 years.

**Figure 1:**
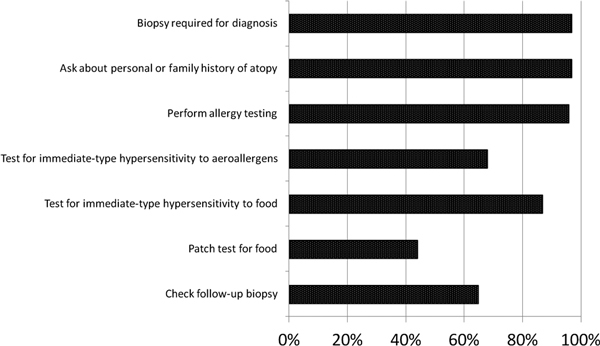
**Responses regarding EoE evaluation**.

The overwhelming majority (97%) responded that esophageal biopsy is required to diagnose EoE, and the same amount do ask about a personal and family history of atopy (Figure 1). In addition, 96% of respondents usually or always recommend allergy evaluation via testing. As for the type of allergy testing, 68% recommend testing for immediate-type hypersensitivity to aeroallergens and 87% recommend such testing for foods. However, the minority (44%) recommend testing for delayed-type hypersensitivity via patch testing for foods. The majority (65%) responded that they do recommend a follow-up biopsy after the initial therapy.

**Figure 2:**
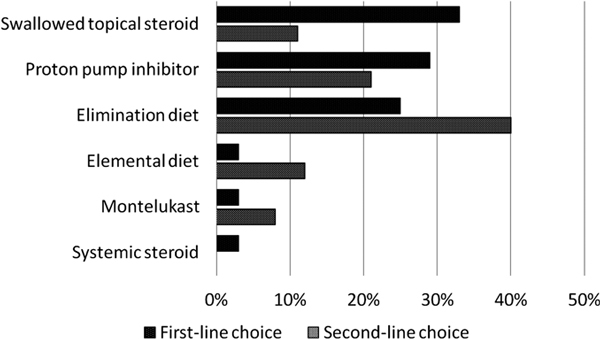
**Responses regarding EoE treatment**.

Most respondents (85%) reported that their patients are on PPIs at initial presentation. The initial therapy prescribed by the respondents was quite variable (Figure 2). One third of respondents reported starting with swallowed aerosolized steroid, such as fluticasone, budesonide, or beclomethasone. Others start treatment with a PPI (29%) or an elimination diet (25%). Systemic corticosteroids, montelukast, and elemental diet (amino acid-based formulas) were the initial therapy offered by only 3% of respondents. The second-line therapy recommended by respondents was also variable. While 40% use elimination diet, 21% use PPIs, 12% use elemental diet, and 11% use swallowed aerosolized steroids as second-line therapy.

**Figure 3:**
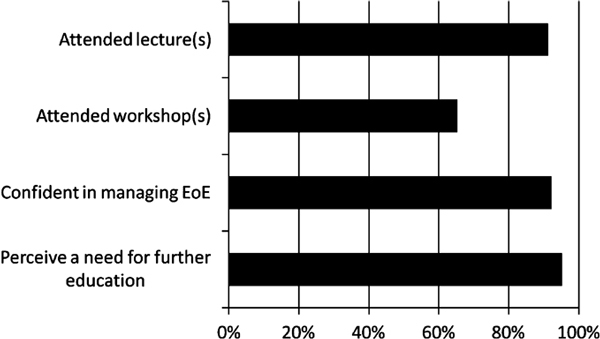
**Responses regarding EoE education**.

Most respondents (91%) reported attending at least one lecture on EoE within the past 3 years (Figure 3). This includes the 58% of respondents who reported attending 3 or more EoE lectures in this time frame. Meanwhile, 65% attended at least one workshop on EoE and one third of respondents attended multiple. Of these, 35% of respondents specifically attended a workshop involving patch testing for foods. Additionally, 19% attended a workshop on elemental diets, and one third attended a workshop on food elimination diets. Workshops on food allergies were attended by 59% of respondents.

Only one fourth of respondents felt completely confident in their ability to manage EoE. Although two thirds of respondents felt somewhat confident, 8% were not at all confident in their management ability. More than 95% of respondents felt a perceived need for further education regarding EoE. Opinions were mixed regarding the preferred format for the further education. While 48% preferred a workshop, 41% preferred a lecture.

When comparing respondents from the United States versus international practitioners, US practitioners recommend follow-up biopsy more frequently (69 vs 50%, *P *= 0.011). US practitioners also recommend immediate-type hypersensitivity to foods more frequently (91 vs 76%, *P *= 0.01). There were no significant differences between genders. Similarly, the number of years in practice did not significantly alter any practice patterns.

Comparing primary specialties, pediatricians were less likely to test for immediate-type hypersensitivity reactions to food (80 vs 96%, *P *= 0.02). Practitioners in private practice were more likely to recommend testing for immediate hypersensitivity reactions to food (92 vs 81%, *P *= 0.03). There was a trend toward performing patch testing for foods among practitioners in rural compared with urban/suburban areas (73 vs 42%, *P *= 0.06). No differences were seen when stratifying by overall patient volume.

Practitioners who see EoE patients more frequently are more likely to ask about a personal or family history of atopy. They are also more likely to test for immediate-type hypersensitivity to aeroallergens (80 vs 53%, *P *= 0.003) and foods (97 vs 82%, *P *= 0.05). Furthermore, those seeing more EoE patients per week are more likely to check a follow-up biopsy after initiating treatment (75 vs 54%, *P *= 0.04). Those practitioners who see more EoE patients tended to be more confident managing EoE (100 vs 85%, *P *= 0.07), although this missed statistical significance.

**Figure 4:**
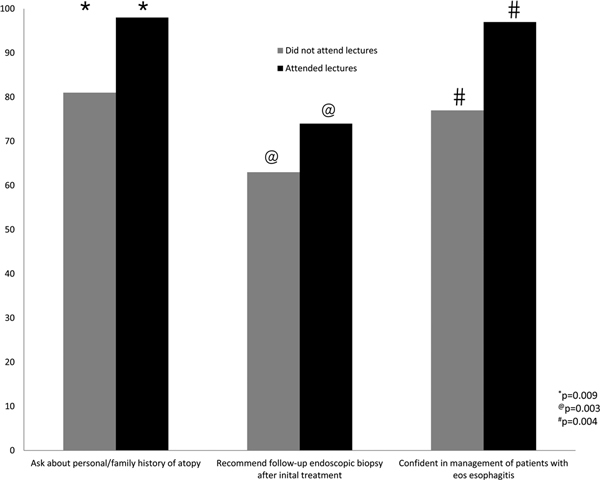
**Association between attending lectures and practice patterns**.

**Figure 5:**
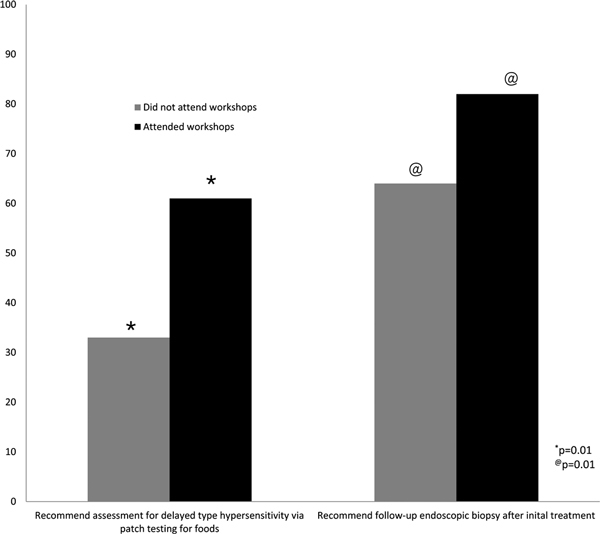
**Association between attending workshops and practice patterns**.

Several differences were seen based on education. Those who attended more lectures were more likely to ask about family or personal history of atopy (98 vs 81%, *P *= 0.009) and to recommend a follow-up biopsy (74 vs 62%, *P *= 0.003). Those who attended more lectures were also more likely to test for immediate hypersensitivity to foods, although this missed significance (92 vs 76%, *P *= 0.08). More EoE lecture attendance correlated to greater confidence in personal ability to manage EoE (97 vs 76%, *P *= 0.004) as depicted in Figure 4. Similarly, those with greater attendance at an EoE workshop were more confident, but this missed statistical significance (96 vs 87%, *P *= 0.19). Greater workshop attendance was correlated with an increased likelihood of performing patch testing for foods (61 vs 33%, *P *= 0.01) and recommending a follow-up biopsy (82 vs 64%, *P *= 0.01) as shown in Figure 5.

## Discussion

Our survey reveals that allergists worldwide are involved in the evaluation and management of patients with EoE. It also demonstrates that, for the most part, allergists are following the best available evidence, with practice variability lying in certain areas that need further clarification, particularly treatment strategies.

Care of the EoE patient is a multidisciplinary venture involving different specialties. The role of allergists is particularly important in evaluation and management of patients with this chronic inflammatory disease. In 2007, a panel of experts developed consensus recommendations related to EoE. More recently, in July 2011, an update of the recommendations was generated, which clarified certain areas regarding the general concept of this disease process and the recommendations based on the evidence from the advancing literature in the field. This survey likely reflects the impact of the 2007 recommendations on the allergy practices. The growing educational activities related to EoE in the past few years may have also affected the practice patterns.

Practitioners from the United States constituted the majority of respondents to this survey, followed by practitioners from Europe. This may reflect better access to the survey by these practitioners. It may also show predilection of EoE among individuals in industrialized countries. Surprisingly, we did not have any respondents from Canada compared with the survey conducted by Spergel et al [3].

The authors realize that because of small number of participants from outside the United States in this survey, it is difficult to compare the practice patterns of the US with non-US practitioners. Although we are aware of this, we found differences that are worth considering. Based on this survey, US practitioners are more likely to recommend allergy evaluation by testing for immediate-type hypersensitivity reactions to foods. This finding could suggest that compared with other countries, US allergists are more likely to consider food allergens as the potential pathogenic factor. Because the current guidelines encourage practitioners to test for immediate-type hypersensitivity reactions to foods, this may also reflect the access to the recommendations and educational activities. However, one may argue that it could simply reflect the difference in reimbursement methods or allocation of resources in the United States compared with other countries.

US practitioners are also more likely to recommend follow-up biopsy after initial treatment. This may reflect increased access to practitioners capable of performing EGD with biopsy in the United States. There are suggestions but no clearly established guideline for follow-up biopsy in the consensus recommendations. Therefore, it could be secondary to recommendations during educational activities regarding these practices. Alternatively, the differences may be related to general differences in the culture of medical practice between the United States and other countries. For instance, a more readily available consultation services in the United States may contribute to this finding.

Two aspects of the practices that affected the approach to the patients were frequency and the number of patients seen with EoE. Practitioners who encounter EoE patients more frequently are more likely to recommend tests for immediate-type hypersensitivity reactions to foods and aero-allergens and to recommend follow-up biopsies after treatment. This may be explained by involvement in more educational activities by those who see more patients. Alternatively, those with fewer EoE encounters may have not yet developed a standard approach to the EoE patient because of a paucity of experience.

With regard to the treatment options for EoE, our results are comparable with the results of a larger survey conducted by Spergel et al [3]. The variability seen in the preferred first-line agent of the respondents is most likely because of the lack of a standardized treatment algorithm in the management of EoE. At the present time, a standard algorithm would be difficult, if not impossible, to create because of the lack of well-designed trials comparing different treatments. The majority of controlled trials involve comparing one steroid regimen versus another. Randomized controlled trials comparing different treatment modalities would be greatly beneficial in guiding first-line preferences and would certainly lend themselves to development of a more established algorithm or formal consensus recommendations.

On their large-scale survey, Spergel et al [3] reported that only a minority of all practitioners adhere to the consensus diagnostic criteria. On a survey of gastroenterologists, Peery et al [40] also found that only minority of gastroenterologists follow the recommended diagnostic criteria. Our survey focused on the screening for atopy by obtaining history and testing for sensitization to allergens; however, we did not specifically ask about the diagnostic criteria being used. Spergel et al [3] reported that the younger practitioners were more likely to adhere to the diagnostic criteria. Our survey did not reveal significant practice pattern differences when stratified by the years in practice, but again we did not specifically inquire about the diagnostic criteria being used.

Several differences are seen in the practice patterns of those who have attended educational sessions. Practitioners who attended more lectures were more likely to inquire about personal or family history of atopy, recommend testing for immediate-type hypersensitivity to foods and aeroallergens, and request follow-up biopsies after initial treatment. These individuals also feel more confident managing patients with EoE. Additionally, those who attended workshops were more likely to perform APT. The mere presence of significant differences in practice patterns based on educational activities demonstrates their effectiveness and influence on physician practice. These differences may also imply that the management recommendations that practitioners receive at such activities either are clearer or more complete than the published guidelines. Future surveys directly asking whether the respondents are completely familiar with the contents of the most recent consensus recommendations along with inquiry into educational activity participation may provide stronger insight into this finding.

Some of the specific differences seen based on the participation in educational activities reside in areas that were not definitively addressed in the 2007 consensus recommendations. For instance, the higher rate of recommending APT among those who more often participate in workshops needs particular attention. The 2007 consensus recommendations recognized the success of the combination of SPT and APT in identifying the potential foods responsible for symptoms but recommended against its use until further data are available. The 2011 consensus recommendations make no further direct recommendations regarding APT, whether it should be used for elimination diet or for other purposes. Greater workshop attendance also correlated to recommending follow-up biopsies after initiating treatment, which is another topic on which the recommendations are less than definitive. This suggests another area of discordance between the published recommendations and teachings at educational activities.

The trend toward increased confidence managing EoE patients among those having attended more workshops and lectures is not surprising. Whether it is because of an increase in the quality or simply an increase in quantity (repetition and reaffirmation) of their EoE education cannot be interpreted based on the survey data.

Most respondents to the survey agreed that there is a need for increased education. However, most also felt at least somewhat, if not completely, confident in managing EoE patients. This may be interpreted as a confidence in one's management ability based on available evidence and recommendations but a concomitant perceived need for further studies with subsequent education and/or strengthened consensus guidelines.

Our survey has several limitations. The majority of respondents were allergy practitioners from the United States, while the second-most represented country had only 5 respondents. This limited the study in its ability to compare the United States to any particular country or geographic area. However, the variety of other nations represented allowed for a very heterogeneous comparison group, thereby improving generalizability. The survey format of this study lends itself to response bias. Therefore, there is the possibility that only those practitioners who have a strong interest in EoE responded to the survey. However, 40% of respondents reported only encountering an EoE patient every few months. As these physicians lacked a significant cohort of EoE patients, it can be assumed that the respondents were not limited to practitioners with a strong interest in EoE.

A survey sent to the gastrointestinal societies along with allergy societies to compare various aspects of diagnosis, follow-up, and management between gastroenterologists and allergists-immunologists will be worthwhile. Moreover, language-specific surveys for the non-English speaking practitioners across the globe will be extremely useful to compare the variation in practice patterns.

In summary, although the prevalence of EoE is rising, our knowledge of optimal management practices is incomplete. There is a degree of variability regarding EoE management in clinical practice, most notably when comparing practitioners who are stratified based on either the frequency with which they see EoE patients or their involvement in educational activities, such as lectures or workshops. The most striking differences between practitioners fall in the best treatment strategy. Future research studies directly comparing different treatment modalities, and research into treatments targeting specific receptors or molecules, will be promising.

## End Note

Presented as a poster at the 2011 World Allergy Congress, December 4-8, 2011, Cancun, Mexico. It was awarded best poster in the category of food disorders.

## Competing interests

The authors declare that they have no competing interests.
